# Supplementary material for: Pressure-Dependent Structure of BaZrO3 Crystals as Determined by Raman Spectroscopy
Source: Materials (Basel). 2022 Jun 17;15(12):4286. doi: 10.3390/ma15124286 (PMC9228820; doi:10.3390/ma15124286)
Supplement: Supplementary file 1 [file materials-15-04286-s001.zip › materials-1759151-supplementary.pdf]

Pressure-Dependent Structure of $BaZrO_3$ Crystals as Determined by Raman Spectroscopy

Dong-Hyeon Gim [1], Yeahan Sur [1], Yoon Han Lee [1], Jeong Hyuk Lee [1], Soonjae Moon [2], Yoon Seok Oh [3,*] and Kee Hoon Kim [1,4,*]

[1] Center for Novel States of Complex Materials Research, Department of Physics and Astronomy, Seoul National University, Seoul 08826, Korea; loyard@snu.ac.kr (D.-H.G.); yhsur2011@gmail.com (Y.S.); lyh9042@gmail.com (Y.H.L.); jeonghyuklee1508@gmail.com (J.H.L.)

[2] Department of Physics, Hanyang University, Seoul 04763, Korea; soonjmoon@hanyang.ac.kr

[3] Department of Physics, Ulsan National Institute of Science and Technology, Ulsan 44919, Korea

[4] Department of Physics and Astronomy, Institute of Applied Physics, Seoul National University, Seoul 08826, Korea

* Correspondence: ysoh@unist.ac.kr (Y.S.O.); khkim@phya.snu.ac.kr (K.H.K.)

Supplementary Materials

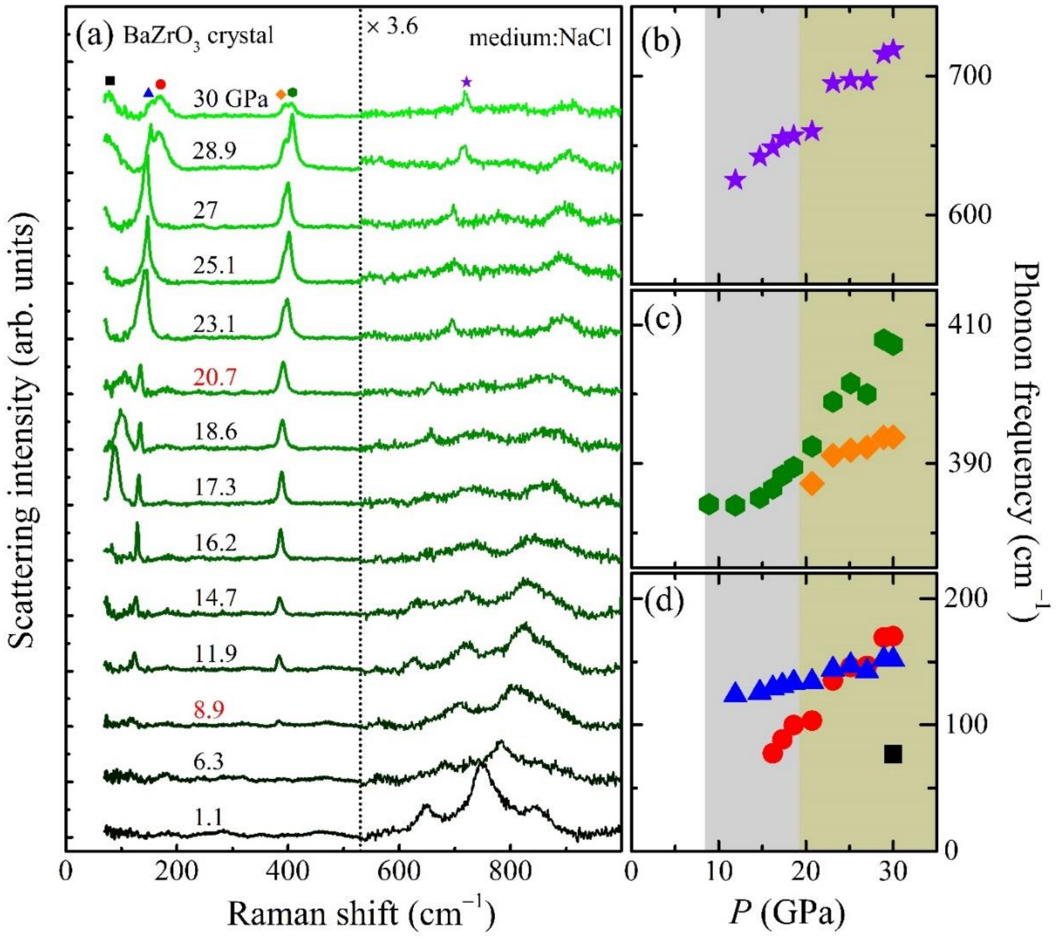

Figure S1: High-pressure evolution of $BaZrO_3$ phonons measured with NaCl pressure medium. (**a**) Pressure-dependent Raman spectra of the $BaZrO_3$ single crystal measured with NaCl pressure medium. Intensities in frequencies above 530 cm^{-1} are magnified by 3.6 for enhanced visibility. The pressures at which a structural phase transition occurs are colored in red characters. (**b**)-(**d**) Evolution of the Raman mode frequencies with variation of pressure obtained from (a) after fittings is presented with the same type of symbols as in (a).
